# Aging research has lost a brilliant investigator—Michael Breitenbach, 1943–2024

**DOI:** 10.1093/femsyr/foaf008

**Published:** 2025-03-07

**Authors:** Ian W Dawes, Terrance G Cooper, Mark Rinnerthaler

**Affiliations:** Ramaciotti Centre for Gene Function Analysis, School of Biotechnology and Biomolecular Sciences, University of New South Wales, NSW 2052, Australia; Department of Microbiology, Immunology and Biochemistry, University of Tennessee Health Science Center, Memphis, TN 38163, United States; Department of Biosciences and Medical Biology, Paris Lodron Universitat Salzburg, 5020 Salzburg, Austria

## Organized chaos

**Figure 1.**
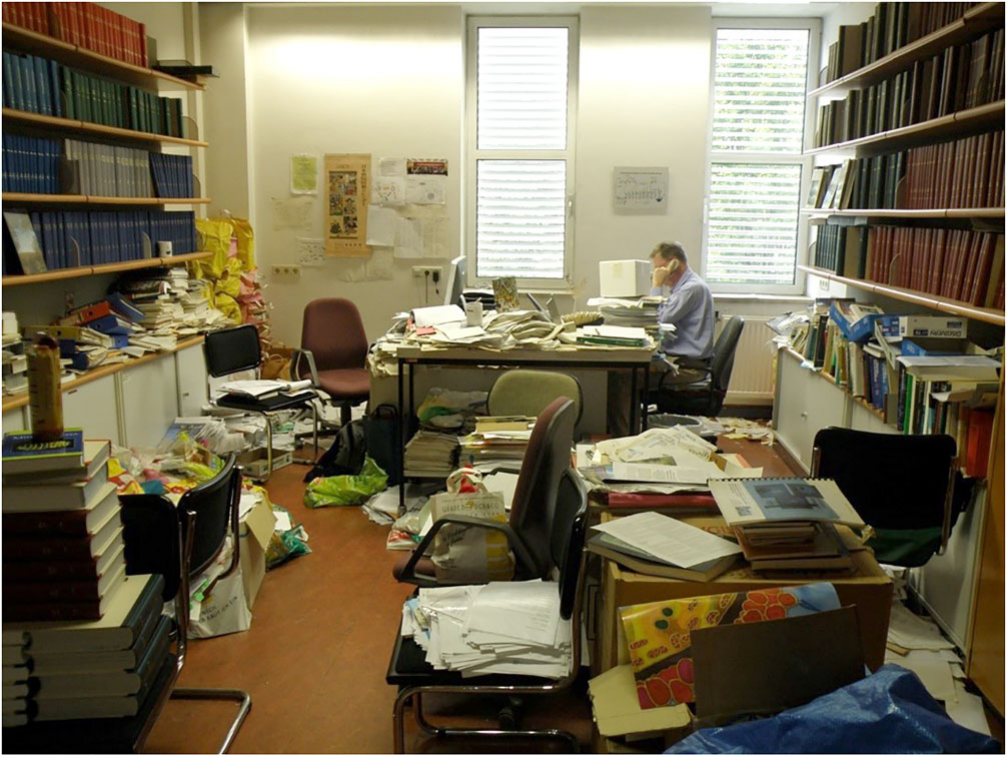
Michael at work in his Salzburg office in 2012, the year he formally retired.

It is with great sadness that we learned of the unexpected death of Michael Breitenbach in October 2024. Michael played a pivotal role in yeast research, contributing not only through his research output but also through substantial service to the disciplines he pursued, generously sharing his time, advice, and materials. Those who knew him will greatly miss his friendship, and his calm, unflappable, and rational demeanour. For those fortunate enough to spend time in Salzburg, Michael and his wife Lore Breitenbach-Koller were always kind considerate hosts, enhancing the experience of a stay in one of Europe’s most attractive cities. They may remember him sitting in his office surrounded by books and papers in what is best described as organized chaos, partly due to his habit of printing out all his emails (Fig. 1).

**Figure 2.**
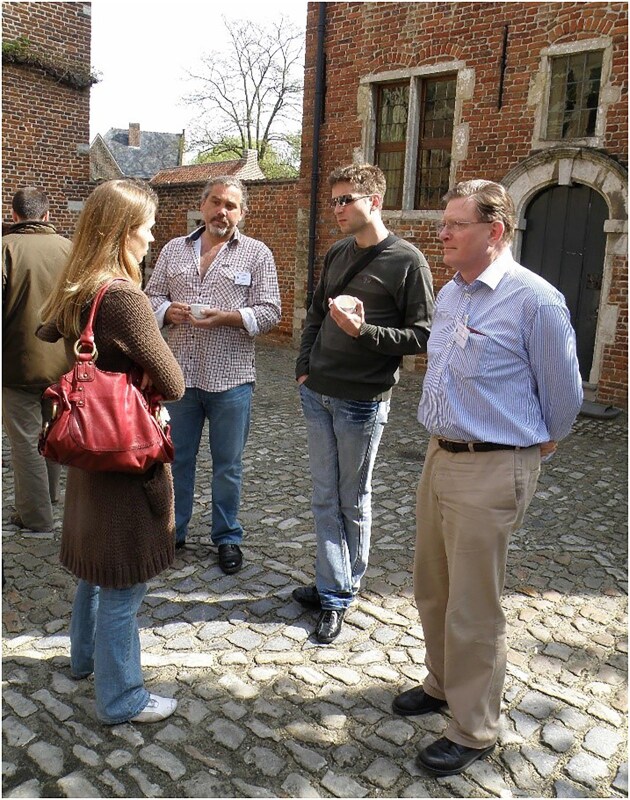
Michael at the 6th International Meeting on Yeast Apoptosis with two of his employees, Mark Rinnerthaler and Peter Laun. The latter had been his Postdoc for nearly a decade.

**Figure 3.**
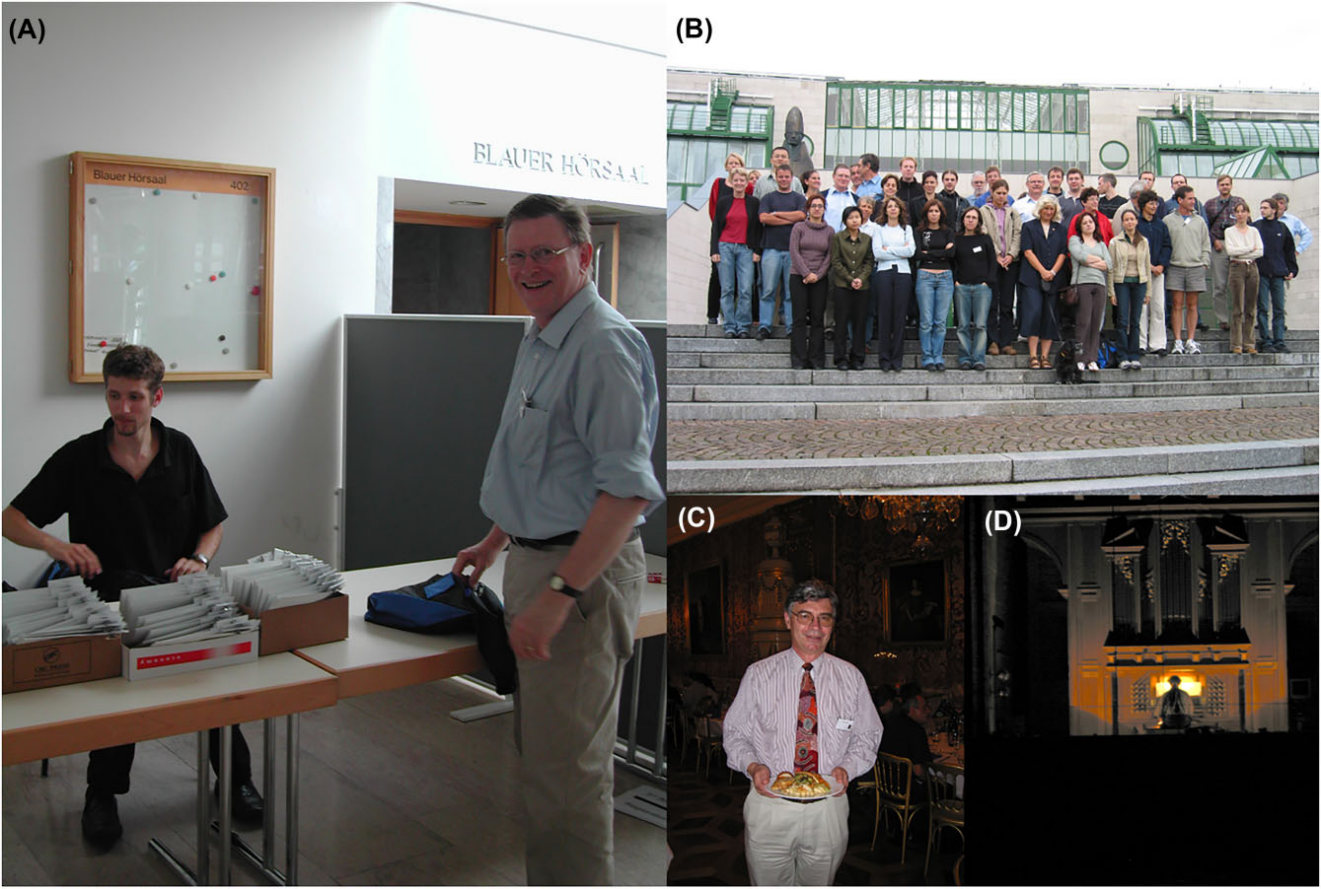
Michael organizing the 3rd International Meeting on Yeast Apoptosis in Salzburg. (A) Michael standing at the reception desk. (B) Group photo of all congress participants, a community that was very close. (C) Ian Dawes at the congress, one of his dearest friends. (D) A special surprise organized by Michael: a church organist playing Bach.

## You best seek another occupation

Michael was born in 1943 during World War II in a small town south of Vienna. His father played in the Viennese Philharmonic Orchestra and Michael told of his father testing him for his musical ability—only to be informed that he should seek some other occupation.

## Those who weathered the university storms with Michael

Michael began his university career in Vienna University studying chemistry and the natural sciences but also with interests in philosophy. Under the supervision of Professor O. Hoffmann-Ostenhof, he investigated *myo-*inositol phosphate metabolism in chicken erythrocytes and graduated with his PhD in 1972. He was appointed as Assistant Professor of Biochemistry at Vienna University under Hoffmann-Ostenhof and later Helmut Ruis. From 1986 to 1991, he was Associate Professor of Genetics in the Department of Microbiology and Genetics under Rudy Schweyen, gaining tenure in 1988. In 1991, he was promoted to Professor of Molecular Genetics at the University of Salzburg. Here, Michael, with his team, built up the Department of Genetics, now the Department of Biosciences and Medical Biology, led by Fritz Aberger, one of the first students to attend Michael’s lectures in Salzburg. Always at his side were Margarethe Zörweg, Elisabeth Kalchschmid, Hans Giesshammer, Thomas Karl, Torsten Klade, and Lore Breitenbach, providing administrative, technical, and scientific support and with whom Michael weathered all storms of university life.

## Learning on the fly

His postdoctoral research included determining the electronic structure of the iron–sulfur cluster in bacterial ferredoxin in the laboratory of Klaus Gersonde in Kiel, and then in Aachen. Returning to Vienna to a position in biochemistry, he came to understand and appreciate the usefulness of *Saccharomyces cerevisiae* as an experimental system and the power of genetic analysis for studying aspects of eukaryotic cell development. Like many of his generation, he learned the techniques of yeast genetics and molecular biology after graduation through courses in yeast genetics, sabbatical visits with Ben Hall and Kelly Tatchell, and at the bench.

## Active to the end

In conjunction with Terry Cooper, Michael wrote a very comprehensive article detailing his research career, revealing his motivation and the outcomes at every stage. This is highly recommended reading since it also encapsulates much of his very rational and philosophical approach to life and his humanity in a clear and readable way (Breitenbach and Cooper 2022). While the rules in the University of Salzburg governing retirement meant that he formally retired in 2012, he continued to work as an Emeritus Professor and was still very active up to the time of his passing—planning updates to his previous reviews on aging and oxidative stress.

## From allergies to dityrosine and development

Michael had a broad range of research interests, not only investigating cell development and aging, but also allergy, contributing to the worldwide first cloning of an allergen (the major birch pollen allergen) as outlined in a highly cited paper (Breiteneder et al. 1989). He suffered from this allergen and understandably had a very dim view of birch trees. In his earlier work with yeast, he made significant contributions to our understanding of sporulation as a differentiation process. With his student Peter Briza, who had discovered dityrosine as a unique component of the spore wall, they exploited a yeast haploid mutant that could produce two-spored asci to identify mutants that were unable to synthesize this late sporulation marker (Briza et al. 1990). This work led them to identify a range of yeast genes relevant not only to dityrosine synthesis but also to others playing important roles in cell development. One of these genes was investigated further by Michael’s colleague Hannelore (Lore) Koller, who subsequently became his second wife, and who now continues research on yeast genetics in her own right as a professor at the University of Salzburg (Chiocchetti et al. 2007).

## Slowing down the aging process

On moving to Salzburg, he shifted his focus from sporulation to oxidative stress, the cellular responses to oxidative stresses, and how they affected aging and apoptosis, which he viewed as special forms of cell differentiation. Michael was a strong proponent of the yeast replicative aging model (see e.g. Laun *et al*. 2006). One of the highlights of this very collaborative research is identifying the potential role of oxidative stress in the aging process as described in the landmark publication by Laun et al. (2001).

His group demonstrated that old yeast mother cells undergo apoptosis triggered by reactive oxygen species originating from the mitochondria. Microarray analysis identified yeast genes differentially expressed in senescent cells, many of which were obtained from apoptotic yeast cells. Systematic phenotypic analysis of these genes revealed a number whose deletion led to oxidative stress resistance and a substantially increased lifespan. One of those genes encodes a mitochondrial ribosomal protein shown to have a second function in growth and aging regulation (Heeren et al. 2009).

Another discovery was the highly conserved gene TCTP (translationally controlled tumour protein), whose product translocates to the mitochondria and the nucleus where it associates with the proteasome and inhibits its activity under heat stress conditions (Rinnerthaler et al. 2006). A third gene (*YNO1*/Ygl 160w) was shown to encode the only nicotinamide adenine dinucleotide phosphate (NADPH) oxidase in *S. cerevisiae*. Superoxide produced by the Yno1 NADPH oxidase may function as a second messenger for growth control regulating the remodeling of the actin cytoskeleton (Rinnerthaler et al. 2012). Overexpression of this gene also causes apoptosis.

## The more, the merrier—generating real impact

Michael’s approach to a problem was to involve as many expert collaborators as possible. He frequently acted as the prime mover of projects involving many international collaborators, organizing the groups to bring each project to a successful conclusion. They included Alena Pichova, Kelly Tatchell, Frank Madeo, Markus Ralser, Ian Dawes, William Burhans, Masayuki Yamamoto, Campbell Gourlay, Dick Dickinson, Jiri Hasek, Dieter Kraft, Arnold Bito, Klaus Richter, Carlo Brusci, and many more. An indication of Michael’s research impact can be seen in the sheer volume of his output, not only papers (>200), reviews, and books but also as a co-inventor of eight key patents in molecular allergology and yeast biotechnology. He continued working right up to the end, even planning further writing.

## Michael the editor

Michael was an Associate Editor for Section Molecular and Cellular Oncology of *Frontiers in Oncology*, Managing Editor of *Frontiers in Bioscience*, and a member of the editorial boards of *International Archives of Allergy and Immunology, Applied and Environmental Microbiology, FEMS Yeast Research, Experimental Gerontology, Mechanisms of Aging and Development*, and *Microbial Cell, Fermentation and Biomolecules*.

## He attended them all

Michael first attended the biannual meetings of the International Conference on Yeast Genetics in 1976 in Schliersee. There he met most of the colleagues and friends with whom he later collaborated. In his own words: ‘*For the next nearly 30 years I have attended every one of the International Conferences on Yeast Genetics and Molecular Biology. What really convinced me to stay with this very active international group of scientists, was: (i) the enormous progress and contributions that yeast groups have made to molecular biology in general, but even more importantly, (ii) the friendly and fair way in which yeast community members treat each other. It has always been easy to get advice, strains, and clones, and I have always responded similarly when asked for advice or materials*’ (Breitenbach and Cooper 2022). Figure 2 shows Michael at the 6th International Meeting on Yeast Apoptosis, enjoying science and colleagues. He was very active in the organization of eight international meetings and congresses, including Chairing the Third International Meeting on Yeast Apoptosis in Salzburg (2004) and the International Aging Research Symposium, Salzburg, held in 2012, the year he formally retired. The former of these meetings was particularly memorable. After dinner, the attendees were ushered into the presence of gods in a darkened cathedral and regaled by the spotlit church organist playing Bach (Fig. 3).

## Michael the colleague—captivation replaces fear as your horizon broadens

Michael’s personality is best described from the perspective of a student who later became a colleague. From a student’s point of view, Michael is best described as a ‘grey eminence’ and ‘wisdom with a dash of chaos’. The first thing that comes to mind is the dreaded, and later loved, Friday afternoon group meetings. These meetings were mostly feared because of their overflowing nature. Michael always led the group in a fatherly, kind, and supportive way. He came very close to the now obsolete term ‘universal genius’, studying almost every subject intensively, be it philosophy, art, literature, or history, and surprising us with the depth of his insights. Group meetings tended to begin with unmoderated scientific questions, only to end up deep in world history or some other unrelated topic. As a PhD student, these in-depth discussions were usually overwhelming, but with growth and development, you became more and more captivated as our horizons broadened.

## Emanating from Michael’s forge

Michael was content only when looking at a subject from multiple angles. Sometimes the experimental controls got really out of hand. His wide-ranging interests were reflected in the common thread of his research. In fact, this thread does not really exist: His interest in many subjects and his general openness to the world were far too great. This complexity of character also influenced his leadership of the group. Students were given a great deal of freedom in their scientific development. This loose rein, only tightened at important steps needed to direct research focus, enabled students to establish their own scientific profiles and develop their own strengths. This interplay of ‘encouragement’ and ‘freedom’ in Michael’s forge shaped a surprising number of students who have gone on to their own academic careers: Lore Breitenbach-Koller, Peter Briza, Heimo Breiteneder, Johannes Farkas, Michael Rützler, Arnold Bito, Renée Schroeder, Andreas Hartig, and Mark Rinnerthaler to name only a few. The latter was Michael’s successor in the field of ‘aging research and stress response’ at the University of Salzburg. Until shortly before his passing, many years after his retirement, the two continued to collaborate in research maintaining the traditional Friday afternoon meetings, always under the guise of science, but always to philosophize about art and the world.

## Our deepest sympathy

Michael is survived by his wife, Lore Breitenbach-Koller, and their two sons Wolfgang and Max, and daughter Jenny from his former marriage to Grete Breitenbach—to all we extend our deepest sympathy.
